# Linking Sleep to Hypertension: Greater Risk for Blacks

**DOI:** 10.1155/2013/436502

**Published:** 2013-04-21

**Authors:** A. Pandey, N. Williams, M. Donat, M. Ceide, P. Brimah, G. Ogedegbe, S. I. McFarlane, G. Jean-Louis

**Affiliations:** ^1^Brooklyn Health Disparities Center, Department of Medicine, SUNY Downstate Medical Center, P.O. Box 1199, 450 Clarkson Avenue, Brooklyn, NY 11203-2098, USA; ^2^Center for Healthful Behavior Change, Division of Internal Medicine, NYU Medical Center, NY, USA; ^3^Division of Endocrinology, Diabetes, and Hypertension, SUNY Downstate Medical Center, NY, USA; ^4^Sleep Disorders Center, Department of Medicine, SUNY Downstate Medical Center, NY, USA

## Abstract

*Background*. Evidence suggests that insufficient sleep duration is associated with an increased likelihood for hypertension. Both short (<6 hours) and long (>8 hour) sleep durations as well as hypertension are more prevalent among blacks than among whites. This study examined associations between sleep duration and hypertension, considering differential effects of race and ethnicity among black and white Americans. *Methods*. Data came from a cross-sectional household interview with 25,352 Americans (age range: 18–85 years). *Results*. Both white and black short sleepers had a greater likelihood of reporting hypertension than those who reported sleeping 6 to 8 hours. Unadjusted logistic regression analysis exploring the race/ethnicity interactions between insufficient sleep and hypertension indicated that black short (<6 hours) and long (>8 hours) sleepers were more likely to report hypertension than their white counterparts (OR = 1.34 and 1.37, resp.; *P* < 0.01). Significant interactions of insufficient sleep with race/ethnicity were observed even after adjusting to effects of age, sex, income, education, body mass index, alcohol use, smoking, emotional distress, diabetes, coronary heart disease, and stroke. *Conclusion*. Results suggest that the race/ethnicity interaction is a significant mediator in the relationship between insufficient sleep and likelihood of having a diagnosis of hypertension.

## 1. Background

In the last two decades, great strides have been made in identifying health disparities in minority populations [1, 2]. Studies have shown an alarming difference in the prevalence of chronic illnesses between blacks and whites, particularly in those that cooccur as in the metabolic syndrome. Epidemiologic data from the National Health and Nutrition Examination Survey (NHANES) have shown that blacks have a greater prevalence of three of the metabolic syndrome's five criteria: hypertension (black: 42.5%, white: 29.1%), diabetes (blacks: 14.6%, whites: 9.9%), and obesity (blacks: 44.1%; whites: 32.4%) [3, 4]. Recognition of these health disparities has led to several important trials targeting reduction of health disparities in these areas. In the context of sleep medicine, little has been done to address metabolic diseases associated with short sleep among racial/ethnic minority groups. 

The observation of a gradual decline in population sleep time along with a concomitant increase in the prevalence of hypertension over the past 20 years provided the impetus to examine whether various racial/ethnic groups might be differentially affected [5, 6]. Recent data has shown that individuals of the black race/ethnicity have a higher prevalence of both short and long sleep durations, relative to their white counterparts [7, 8]. In an earlier investigation, we showed that blacks were twice as likely to report short sleep compared with individuals of the white race/ethnicity [9]. Indeed, the fact that short sleep duration is linked to greater mortality and that it is more prevalent among blacks suggests greater mortality risks associated with short sleep in that population [10, 11].

It is uncertain whether disparities in sleep duration are determined by lifestyle choices or by a greater prevalence of untreated sleep apnea among blacks [9]. Nonetheless, it is evident from recent findings that sleep restriction among blacks might put them at a greater risk for metabolic diseases associated with short sleep [12–14]. The present study investigated whether blacks reporting short or long sleep durations are at a greater likelihood for hypertension, relative to their white counterparts independently of known medical covariates.

## 2. Methods

### 2.1. Participants

A total of 25,352 Americans (age range: 18–85 years) who participated in the 2009 National Health Interview Survey (NHIS) provided sociodemographic and subjective data as well as data regarding physician-diagnosed chronic conditions for the present analysis. They estimated their habitual sleep duration. Analysis focused on race/ethnicity effects on associations between sleep duration and hypertension. Final weights were applied to all analyses to adjust for the use of complex design in the NHIS. 

### 2.2. Procedures

NHIS is an ongoing, cross-sectional, in-person household interview survey conducted annually by the National Center for Health Statistics of the Centers for Disease Control and Prevention. The NHIS uses a multistage area probability design sampling a noninstitutionalized representative of US civilian population. Probability samples of the adult population of all 50 states and the District of Columbia were obtained. The final sample was characterized by a response rate of 69%. As the response rate was relatively low, we compared demographic characteristics between responders and nonresponders, finding no significant differences. Details on sample design can be found in the Design and Estimation for the National Health Interview Survey [15]. 

During face-to-face interviews conducted by trained interviewers from the US Census Bureau, volunteers provided sociodemographic data and information about physician-diagnosed chronic conditions. Self-reported diseases were defined based on the answer “yes” to the question “Have you ever been told by a doctor or other health professional that you have (disease or condition)?” Information was also obtained on the mood of the participants within the last 30 days prior to the interview, for example, feelings of sadness, hopelessness, worthlessness, and poor effort. Using this information, a depression severity score was generated which was a composite score estimated using the K-6 scaling system [16]. Information about the feelings of sadness, hopelessness, worthlessness, and poor effort were used to generate a score ranging from 0 to 24. Scores ≥13 indicated a greater degree of depression—this group was defined as having emotional stress [17]. Information on both weight and height was also collected by the interviewers—this was used to generate BMI. Body mass index was dichotomized as either normal weight (BMI ≤25 kg/m^2^) or overweight/obese (BMI ≥25 kg/m^2^) for descriptive purposes. Chronic conditions included hypertension, coronary heart disease, diabetes, and stroke. Participants also estimated habitual sleep duration (using full hour units, i.e., 5 hours, 6 hours, 7 hours, etc.); no information on specific sleep disorders was elicited during the interview. Participants could only report sleep time in one-hour increments and in whole number units with instructions to round 30 minutes (1/2 hour) or more UP to the next whole hour and dropping 29 or fewer minutes. Habitual sleep duration was coded as either short sleep duration (<6 hours/night) or long sleep (>8 hours/night) with a reference of 6–8 hours/night representing sufficient sleep duration [18]. These cut-off points were chosen based on previous research showing health risk associated with short and long sleep durations [19, 20]. Race/ethnicity was classified according to the revised 1997 US Office of Management and Budget standards for race ethnicity which government agencies follow.

Surveys were conducted using computer-assisted personal interviewing (CAPI), which utilizes a computer program for data collection that guides the interviewer through the questionnaire. The interviewer enters survey responses directly into the computer. The program determines through a computer algorithm whether data entered by the user match against all possible responses to specific questions; the program also checks for consistency against other data collected during the interview and saves the responses into a survey data file [21]. 

### 2.3. Statistical Analysis

Frequency and measures of central tendency were used to describe the sample. In preliminary analyses, Pearson and Spearman correlations were used to explore relationships between variables of interest; only factors showing a *P* value <0.05 were considered in the final regression model [22]. ANOVA was used for group mean comparisons, and Chi-square test was employed to assess differences in categorical variables. The association between sleep duration and hypertension was assessed by logistic regression: adjusted and unadjusted analyses. Hypertension was considered the dependent variable in this analysis (a binary measure where the participants had self-reported hypertension versus no hypertension), sleep duration was assessed as a categorical variable, and participants were categorized into the aforementioned groups. Univariate analyses were first carried out followed by multivariate analyses. In these analyses, adjustment was made for sociodemographic factors (age, gender, education, income, and obesity), risk factors (smoking, alcohol use, and physical inactivity), and medical comorbidities (emotional distress, coronary heart disease, diabetes, and stroke). The factors included in the final and most parsimonious model were selected based on presence of a known relationship with sleep disturbances from the extant literature and our preliminary analysis. Statistically significant variables with a *P* value <0.01 on univariate analysis were carried forward in a stepwise multivariate logistic regression analysis. Race/ethnicity was tested as a stand alone variable in our initial models. We then tested for possible effect modification by race/ethnicity of the association between sleep duration and self-reported hypertension by interaction models as outlined in Jaccard [23]. All analyses were performed with SPSS, version 20.0, using sampling weights published for the respective cohort of NHIS.

## 3. Results

A total of 25,352 Americans (age range: 18–85 years) enrolled in the 2009 NHIS provided complete data for this analysis. Of the sample, 82% self-reported their race/ethnicity as white and 18% as black. Among the respondents, the average age was 46.3 years and 52.0% were female.

Sociodemographic and health characteristics of study participants are provided in Table 1. Overall, blacks were younger than their white counterparts; blacks were more likely to be female, less likely to have finished high school, considerably less likely to report income of greater than 35,000, less likely to be of normal weight (BMI <25), less likely to report alcohol use and smoking, more likely to report sedentary lifestyle, and reported greater levels of emotional distress. Black participants were more likely to report a diagnosis of diabetes and hypertension, whereas whites had a higher prevalence of heart disease. Of the sample, 29% of white participants and 35% of black participants reported that they have been told by a healthcare professional that they have hypertension. Blacks tended to report more insufficient sleep while a significant percentage of whites reported sleeping 6–8 hours (*P* < 0.001). 

As shown in Table 2, logistic regression analysis among black and white participants showed that short sleepers had a higher likelihood of reporting a diagnosis of hypertension compared with those sleeping 6–8 hours (OR = 1.21, 95% CI = 1.04–1.41). In our model, alcohol use and physical activity were statistically insignificant. Age and sex did not have a significant influence, but race/ethnicity and medical comorbidities had significant contributions in explaining observed odds ratios. Obesity and diabetes were the main drivers of the relationship in both short and long sleep durations. 

Table 3 presents adjusted and unadjusted odds ratios of the likelihood of reporting hypertension by testing interactions of race/ethnicity with insufficient sleep durations. In our hierarchical model exploring black and white race/ethnicity, we observed significant interactions of race and ethnicity with both short and long sleep in all models. Black short and long sleepers were more likely to report hypertension compared with respondents reporing habitual sleep duration of 6–8 hours (see Table 3). Education and obesity were significant contributors to the interaction of insufficient sleep with race/ethnicity. Further adjustments for medical factors indicated marginally yet statistically significant relationships for long sleep. The results of the final regression model for each confounder are presented in Table 4. Post-hoc analysis using continuous BMI and categorization of overweight and/or obese in dummy coded variable did not change the overall model results with respect to significance or magnitude (not shown).

## 4. Discussion

Recent evidence indicates that individuals sleeping abnormally less or more than the population average sleep duration are at greater risk of cardiovascular disease [24, 25]. The main finding of our analyses is that adult short and long sleepers had a greater likelihood of hypertension and the relationship varied by race/ethnicity. Blacks who reported sleeping habitually <6 hours or >8 hours per night were characterized by a higher likelihood of hypertension compared with their white counterparts. Of note, the race/ethnicity effect was independent of known sociodemographic and medical confounders for long sleep.

Our finding of greater likelihood of hypertension among insufficient sleepers is consistent with previous data suggesting that insufficient sleep durations predicted increased odds of incident hypertension among American adults, both age related and unrelated. In addition, they are also consistent with recent epidemiologic studies that have shown associations between sleep duration and hypertension [26, 27]. 

Our investigation, with a representative adult population of the United States, examined the potential effects of race/ethnicity on associations between sleep duration and hypertension. These findings are consistent with the CARDIA study, an investigation examining cross-sectional and longitudinal associations between objectively measured sleep duration and blood pressure in middle-aged adults. In the CARDIA study, sleep duration partially mediated the larger increases in blood pressure that are associated with African-American race, particularly when diastolic blood pressure was considered [28]. While our analyses focused on black and white Americans, we should note that reduced sleep time may contribute to the severity of hypertension in other racial groupings [29]. It has been shown that short sleep has been independently associated with hypertension among young and middle-aged Korean adults [30].

Prevalence of hypertension and its subsequent sequelae have been shown to be higher among blacks relative to whites [31, 32]. Coding single equally Chi polymorphisms, albeit rare and inadequately understood, have been identified to influence essential hypertension [33]. Recent literature has shed light on the impact of race/ethnicity through social and environmental elements in the epidemiological study of hypertension [34]. Among the pre-hypertensive cohorts from the REGARDS study, blacks were at higher risk for both hypertension and prehypertension than whites with conventional risk factors such as obesity and excess alcohol consumption acting as lead modifiers [35]. In our investigation, obesity was also a significant contributor in our models. Investigators from the multicenter Multi-Ethnic Study of Atherosclerosis (MESA) attempted a novel approach beyond the conventional risk factors to study neighborhood characteristics such as violence and disorder to examine racial/ethnic differences among hypertensives. They found that neighborhood stressors contributed significantly to the positive association between blacks and hypertension [36].

To date, there are inconclusive explanations for the increased prevalence of hypertension among insufficient sleepers. Thus, further investigations aiming at better understanding of the mechanistic link is warranted. Probable mechanisms elucidating increased blood pressure among patients with short sleep may be linked to sustained activation of the sympathetic nervous system [37, 38]. Another possible explanation relates to the process or the dipping phenomenon occurring during slow-wave sleep [39]. Although studies in sleep-deprived animals [40] and humans [41] have shown a strong physiological association between sleep and hypertension, more clinical trials need to be performed to delineate the underlying pathophysiology. Previous research has shown that long sleep is associated with hypertension [42], obesity [43], and stroke [44], and long sleep may actually be more detrimental to health than short sleep. Underlying mechanisms that could be responsible for the effect of long sleep, as suggested in previous analyses, include sleep fragmentation and photoperiodic abnormalities, which could all lead to increased blood pressure levels [45]. Studies have found higher prevalence of systemic hypertension among those with sleep fragmentation possibly related to transient elevation mainly due to frequent arousal and transient increase in BP and sympathetic activity due to these arousals. The observed association of long sleep with hypertension needs to be corroborated by future studies before definitive conclusions can be reached. It is also evident that both long and short sleep durations are associated with elevated markers of inflammation, abnormal lipid profile, insulin resistance, increased BMI, and diabetes mellitus, all independent predictors of hypertension. 

We surmise that sleep duration may be a risk factor in the development of hypertension in addition to insulin resistance [46], obesity [47, 48], and diabetes [49, 50]. Among racial/ethnic groups, African-American adults have the highest rates (44%) of hypertension [51] and blacks have greater prevalence of short sleep and sleep disturbance compared with whites [7, 9, 52]. While the exact causes of sleep loss were not ascertained in these studies, conceivably it might result from untreated sleep-related disorders such as sleep apnea, which is known to affect blacks disproportionately [11]. In sum, findings of our study suggest that individuals reporting insufficient sleep durations and who are characterized by a diagnosis of hypertension may constitute a vulnerable population, requiring optimal clinical management to reduce cardiovascular sequelae from hypertension.

Our study has some notable limitations. One such limitation relates to our inability to verify reported sleep durations and clinical diagnoses. However, previous self-reported data from blacks and whites in a smaller cohort showed no significant differences between self-reported and actigraphic sleep durations [53]. Another important limitation concerns the unavailability of data regarding the presence of sleep apnea, which would have provided some explanation of the associations between short sleep and hypertension. There is also the possibility of potential of misclassification because of undiagnosed hypertension by healthcare professionals. Our analysis did not adjust for other important factors such as sleep apnea, insomnia, and excessive daytime sleepiness or fatigue, which have adverse effects on sleep duration; such data were not available in the NHIS data.

Our study explores the relationship between race and ethnicity and hypertension and does not establish any cause and effect relationships between race/ethnicity, sleep durations, and likelihood of reporting hypertension or exclude reverse causality because of the cross-sectional design.

It is of interest to examine whether our observations could be replicated in other nationally representative data and clinical trials. Notwithstanding these limitations, our results provide some insight as to the importance of race/ethnicity in understanding associations between sleep durations and likelihood of hypertension.

## 5. Conclusions

There is a greater likelihood of hypertension for blacks with insufficient sleep duration compared with whites. Assessment of sleep duration should be performed in all hypertensives, especially among those of the black race/ethnicity.

## Figures and Tables

**Table 1:** Characteristics of adult participants from the National Health Interview Survey (NHIS). Stratified by race/ethnicity; *P* value obtained from *λ* square.

	Black	White	*P* value
Participants (*N*; %)	4571; 18.0	20781; 82.0	
Age (mean)	43.3	46.8	
Female sex (%)	55.6	51.1	<0.001
Education; ≥HS (%)	89.2	92.1	<0.001
Income; ≥35,000 (%)	53.1	71.1	<0.001
Body mass index (%; normal weight; ≤25 kg/m^2^) (%)	27.9	35.7	<0.001
Alcohol use (%)	71.3	82.7	<0.001
Smoking (%)	35.5	44.7	<0.001
Physical activity (%)	52.0	61.9	0.008
Emotional distress (%)	3.5	2.5	<0.001
Diabetes (%)	12.1	8.8	<0.001
Heart disease (%)	6.4	8.4	0.004
Stroke (%)	3.3	2.7	0.045
Short sleepers (<6 hours) (%)	12.3	7.3	<0.001
Long sleepers (>8 hours) (%)	11.1	10.0	<0.001
Hypertension (%)	35.3	28.5	<0.001

**Table 2:** Multivariate-adjusted logistic regression analysis indicating odds ratios (ORs) associated with the presence of hypertension between short and long sleep durations in the selected population. In the model, short sleep was defined as sleep durations <6 hours and in Model B; long sleep was defined as sleep durations >8 hours; reference sleep was 6 to 8 hours.

Variables	Short sleep (<6 hours)				Long sleep (>8 hours)			
	*P*	OR	95% CI		*P*	OR	95% C.I.	
			Lower	Upper			Upper	Lower
Sleep duration (categorized)	0.001	1.21*	1.04	1.41	0.834	1.02	0.88	1.18
Age	<0.001	1.06	1.06	1.06	<0.001	1.06	1.05	1.06
Sex (reference: male)	0.001	0.87	0.87	0.96	<0.001	0.89	0.81	0.97
Race (reference: white)	<0.001	1.68*	1.47	1.91	<0.001	1.74*	1.53	1.97
Income (reference: <$35,000)	0.004	0.90	0.81	0.99	0.10	0.94	0.85	1.03
Education (reference: high school)	0.001	0.83	0.71	0.95	0.12	1.13*	0.99	1.30
Obesity (reference: nonobese)	<0.001	2.38*	2.17	2.61	<0.001	2.45	2.23	2.69
Alcohol (reference: never)	0.34	1.07	0.93	1.22	0.51	1.03	0.91	1.16
Smoking (reference: never)	0.001	1.12*	1.02	1.22	<0.001	1.17*	1.07	1.28
Activity (reference: no physical activity)	0.74	1.02	0.92	1.12	0.93	1.02	0.93	1.13
Emotional Distress (reference: none)	<0.001	1.83*	1.35	2.48	<0.001	1.97*	1.43	2.70
Diabetes (reference: none)	<0.001	3.15*	2.69	3.70	<0.001	3.40*	2.95	3.93
Coronary heart disease (reference: none)	<0.001	2.06*	1.76	2.42	<0.001	2.03*	1.74	2.36
Stroke (reference: none)	<0.001	2.08*	1.58	2.73	<0.001	1.97*	1.48	2.62

*Variables contributing significantly to the relationship.

**Table 3:** Multivariate-adjusted hierarchal logistic regression analysis indicating odds ratios (ORs) associated with the presence of hypertension based on interactions between short/long sleep duration and black and white race/ethnicity. Model adjustments were Model 1 adjusted for age, and sex; Model 2 adjusted for Model 1 + education, income, alcohol use, smoking, physical activity, and body mass index; Model 3 adjusted for Model 2 + emotional distress, diabetes, coronary heart disease, and stroke.

Variables	Short sleep (<6 hours)					Long sleep (>8 hours)			
	*P*	OR	95% CI		Sufficient Sleep (6–8 hours)	*P*	OR	95% CI	
			Upper	Lower				Lower	Upper
Sleep∗Race									
Unadjusted	<0.001	1. 34^¥^	1.02	1.75	1.0 (Referent)	<0.001	1.37^¥^	1.07	1.75
Model 1^a^	<0.001	1.30^¥^	0.97	1.74	1.0 (Referent)	<0.001	1.12^¥^	0.83	1.50
Model 2^b^	<0.001	1.08^¥^	0.81	1.45	1.0 (Referent)	<0.001	1.01	0.74	1.38
Model 3^c^	<0.001	0.95	0.71	1.28	1.0 (Referent)	<0.001	1.03	0.74	1.43

^*¥*^Values significant than 1.0.

^1^Reference category is white for race/ethnicity and habitual sleep duration of 6 to 8.

**Table 4:** Multivariate-adjusted logistic regression analysis indicating odds ratios (ORs) for the presence of hypertension associated with short/long sleep duration among black and white participants.

Variables		Short sleep (<6 hours)				Long sleep (>8 hours)		
	*P*	OR	95% CI		*P*	OR	95% CI	
			Lower	Upper			Lower	Upper
Sleep duration∗race/ethnicity (categorized)	0.001	0.95	0.71	1.28	<0.001	1.03	0.74	1.45
Age	<0.001	1.05	1.02	1.21	<0.001	1.04	1.01	1.09
Sex (reference: male)	0.001	0.87	0.80	0.96	0.01	0.89	0.81	0.97
Income (reference: <$35,000)	0.004	0.90	0.82	1.00	0.17	0.94	0.85	1.03
Education (reference: high school)	0.001	1.21*	1.05	1.39	0.08	1.13*	0.99	1.30
Obesity (reference: nonobese)	<0.001	2.38*	2.17	2.62	<0.001	2.45*	2.23	2.69
Alcohol (reference: never)	0.34	1.07	0.93	1.22	0.68	1.03	0.91	1.16
Smoking (reference: never)	0.001	1.12*	1.02	1.22	<0.001	1.17*	1.07	1.28
Activity (reference: no physical activity)	0.74	1.02	0.93	1.12	0.62	1.02	0.93	1.13
Emotional distress (reference: None)	<0.001	1.83*	1.35	2.48	<0.001	1.97*	1.43	2.70
Diabetes (reference: none)	<0.001	3.15*	2.69	3.70	<0.001	3.41*	2.95	3.93
Coronary heart disease (reference: none)	<0.001	2.06*	1.76	2.42	<0.001	2.03*	1.74	2.36
Stroke (reference: none)	<0.001	2.09*	1.59	2.74	<0.001	1.97*	1.48	2.62

*Variables contributing significantly to the relationship.
